# 

**Published:** 1900-01-27

**Authors:** 


### NOTICE TO CORRESPONDENTS.

All MS., Letters, Books for Review, and other matters intended for the Editor should be addressed THE EDITOR, "THE HOSPITAL," THE HOSPITAL BUILDING, 28 & 29, SOUTHAMPTON STREET, STRAND, W.C.

The Editor cannot undertake to return rejected MS., even when accompanied by stamped directed envelope.

### Notice to Advertisers and Subscribers.

All Advertisements, Orders for Copies of THE HOSPITAL, and Business Communications should be addressed to **THE MANAGER (Not to the Editor)**, THE HOSPITAL, The Hospital Building, 28 & 29, Southampton Street, Strand, London, W.C.

The Cost of Subscription, post free, is as follows:—Per week, 2½d.; per quarter, 2s. 8d.; per half-year, 5s. 3d.; per annum, 10s. 6d. Foreign:—Per annum, 15s. 2d., payable, in all cases, in advance.

**NOTICE.—Vol. XXVI. of "The Hospital" (April, 1899, to September, 1899), in handsome cloth binding, is now on sale at the Office of this Journal, price 7s. 6d. Cases for binding the Numbers contained in this Volume 1s. 6d. Index to Vol. XXVI., 2½d., post free.**

**The Monthly Part for February will be ready on 1st February. Price 6d., by post 8d.**

### SCRAPS AND GLEANINGS.

THE Dewsbury Joint Hospital Board have agreed to accept the amended tenders for the new building, the total of which amounts to £22,675.

MESSRS. CHIVERS AND SONS, of Histon, Cambridge, are presenting to the War Office for the use of our sick and wounded in South Africa 5,000 packets of their jelly.

THE total of the amount contributed during the last twenty-nine years to the Liverpool Hospital Sunday and Saturday Funds is £295,666, of which total £198,393 has resulted from Hospital Sunday efforts.

To help clear off last year's deficit in the income of the Dundee Royal Infirmary Mr. Armitstead has generously given a further sum of £500, devoid of conditions. There is still a deficit of £1,300, which it is hoped may soon be wiped off.

IN response to the appeal of the Mayor, the committee of the Bath Homœopathic Hospital have decided to place two private wards at the disposal of commissioned officers invalided from the war, and four beds for the use of non-commissioned officers and men.

THE income for the past year of the Dewsbury and District General Infirmary amounted to £2,210, of which £1,077 represented Hospital Saturday collections and £158 Sunday collections. The board have offered four beds in the Dewsbury Infirmary for the use of wounded soldiers.

AT a recent meeting of the Ventnor Isolation Hospital Committee it was proposed that an iron building should be erected, on the ground that a more expensive building was not required. This proposal was, however, not carried, and it was decided to have a more elaborate hospital, costing £1,000, to £1,500.

THE hospital for the reception of scarlet fever patients which the district committees are building at Burghmuir is nearing completion, and a conference of the representatives of the district committees of the Perth Local Authority and of the directors of the Perth Infirmary has been held to arrange for the reception of patients.

THE yearly report of the Bath Homœopathic Hospital shows that there has been a slight increase in the payments of private patients, but the subscriptions showed a serious falling off, which will considerably cripple the work of the institution unless further support is forthcoming. During the past year the number of out-patients was 8,589.

IN view of the proposal to spend £5,000 on extensions to the Aughton Isolation Hospital, the details of the working given by the medical superintendent at the recent quarterly meeting of the Joint Hospital Committee are interesting. They show that the accommodation for enteric fever is too small, and also that there should be more accommodation for scarlet fever.

IT has been decided to build a laundry in connection with the Lincoln County Hospital, by which means the hospital will be brought up to date in the matter of heating arrangements as the boilers for use in the laundry will supply all that is necessary in that respect. The estimated extra cost of this addition is £132 per annum, while the buildings, fittings, &c., are to cost just over £5,000.

DURING the past year the income of the Lincoln County Hospital was £5,089, as against £5,114 the previous year.

BY direction of H.R.H. the Princess of Wales a cheque for £7 19s. 2d. has been received by the secretary of the British Home and Hospital for Incurables, Streatham, S.W., as the result of a further sale of Canon Fleming's sermon, "Recognition in Eternity." The British Home and Hospital for Incurables was the first charitable institution in England to receive the honour of Her Royal Highness's patronage.

AT a recent meeting of the Warrington Town Council the resolutions of the Health Committee were accepted after some discussion. They were to the effect that the tenders for the extensions at the infectious diseases hospital, amounting altogether to £9,606, should be accepted; and that the Town Clerk be instructed to apply to the Local Government Board for authority to borrow £5,500 for the purposes of hospital extension.

ON account of the war in South Africa the Committee of Management of the Royal South Hants Infirmary have decided to refrain from holding their annual ball this year. They, however, suggest that those who under ordinary circumstances would have taken tickets for the ball should give a special donation to the funds of the infirmary. They point out that as their expenditure for 1899 will have exceeded their income, as usual, by many hundreds of pounds, they cannot afford to lose the £50 or £60 which would have accrued from the subscriptions to the ball.

FROM the circular issued by the governors of the General Hospital of Tunbridge Wells regarding the enlargement of the hospital it will be seen that for the Children's Jubilee Hospital (which will be specially built with a separate entrance) £3,000 has already been subscribed. Consequent on the great increase of population in the town, the governors have also been considering the necessity of providing additional accommodation for out-patients, and for this purpose another £3,000 will be required, and the committee are appealing to the public for further contributions.

AT the monthly meeting of the Asylum Infirmary Board of Montrose it was stated by the medical superintendent that the number of patients remaining on the register on December 31st, 1899, showed a decrease of sixteen as compared with those on the register on the corresponding date of the previous year. At the same meeting it was resolved to privately discuss the matter of a petition regarding the exemption of hospitals and infirmaries from local rates, which subject had been brought to their notice in a communication received from the secretary of the Central Hospital Board.

AT the recent quarterly meeting of the governors of the Leicester Infirmary the Chairman stated that last year's income amounted to £11,932 odd, and the expenditure to £12,322 odd, showing an increase of expenditure over income of £389 odd. In addition to this there was a special expenditure of £635 4s. 7d. in regard to building, so that the total excess of expenditure over income was £1,015 2s. 4d. Although the legacies for the year were somewhat smaller than previously, this did not affect the conspicuous deficit. The annual collections at the churches and chapels also showed a decrease on the previous year; but from the Saturday collections the Committee had received £391 in excess of the mount collected from this source last year.

### THE STUDENT OF MEDICINE.

### APPOINTMENTS.

E. BRAMWELL, M.B.Edin., appointed Senior House Physician to the National Hospital for Paralysed and Epileptic. E. F. Buzzard, M.A., M.D.Oxon., M.R.C.P., appointed Junior House Physician to the National Hospital for the Paralysed and Epileptic. A. Dowling, L.R.C.P., L.R.C.S.Edin., appointed Medical Officer for the Haycock District of the Warrington Union. Laura Forster, M.D.Berne, L.R.C.P.&S.Edin., appointed Medical Officer to the Cutler Boulter Provident Dispensary, Oxford. M. B. James, M.B., Ch.B.Vict., appointed Assistant Medical Officer of the St. Mary Islington Workhouse and Infirmary. H. Leader, M.B., appointed House Physician to the East London Hospital for Children, Shadwell, E. C. B. Rossiter, F.R.C.S.Edin., &c., appointed Clinical Assistant to St. John's Hospital for Diseases of the Skin, Leicester Square. E. S. Saunders, M.D., C.M.Aberd., appointed Medical Officer to the Dispensary, Royal Albert Hospital, Devonport. T. E. Saxby, L.R.C.P., L.R.C.S.E., &c., appointed Medical Officer of Health for the District of Unst, Shetland, N.B.

Crown 8vo., cloth, 2s. 6d.

**HOSPITAL EXPENDITURE: THE COMMISSARIAT.**

**Reprinted from THE HOSPITAL.**

"This should prove a valuable handbook to those upon whom rests the ressonsibility of hospital finance, and should be of interest to the public as giving a fair idea of what this department should and does cost."—*Manchester Courier.*

LONDON: THE SCIENTIFIC PRESS, Ltd., 28 & 29, Southampton Street, Strand, W.C.

### NOW READY,

A REPRINT OF

### A NURSING HANDBOOK

TOGETHER WITH S. MAW, SON, AND THOMPSON'S

### COMPLETE CATALOGUE OF NURSING REQUISITES.

The above work is now republished at a cost of 1s. per copy. Messrs. S. M., S., and T. have been compelled to make this charge on account of the extreme popularity of the work and the great expense incurred in its production.

***Post Free on receipt of 1s.***

Nurses' Bags.

### S. MAW, SON, and THOMPSON,

7 to 12, ALDERSGATE STREET, LONDON, E.C.

